# Association for research on biosignaling and communication first world conference on cellular communication and signaling

**DOI:** 10.1002/ccs3.12048

**Published:** 2024-08-13

**Authors:** Bernard Perbal, Ralf Weiskirchen

**Affiliations:** ^1^ International CCN Society Nice France; ^2^ Institute of Molecular Pathobiochemistry Experimental Gene Therapy and Clinical Chemistry (IFMPEGKC) RWTH University Hospital Aachen Aachen Germany

**Keywords:** ARBIOCOM, cell‐cell communication, cooperation, data sharing, interacting, network, scientific community, scientific societies, signaling

## Abstract

The present manuscript reports on the progress made toward the official announcement of the first World Conference on Cellular Communication and Signaling. This conference is made possible by the Association for research on biosignaling and communication initiative, which was originally launched in 2020 and revitalized during the 12th International Workshop on the Cell Communication Network family of genes in Oslo (June 20–23, 2024). The aim of this conference is to facilitate interactions among the members of societies interested in all aspects of research on Biosignaling and Communication. It is intended to provide a platform for collaborative efforts aimed at unraveling and understanding the functioning of biological pathways in both normal and pathological conditions.

## Graphical abstract

Schematic complementary interactions in cell communication and signaling.

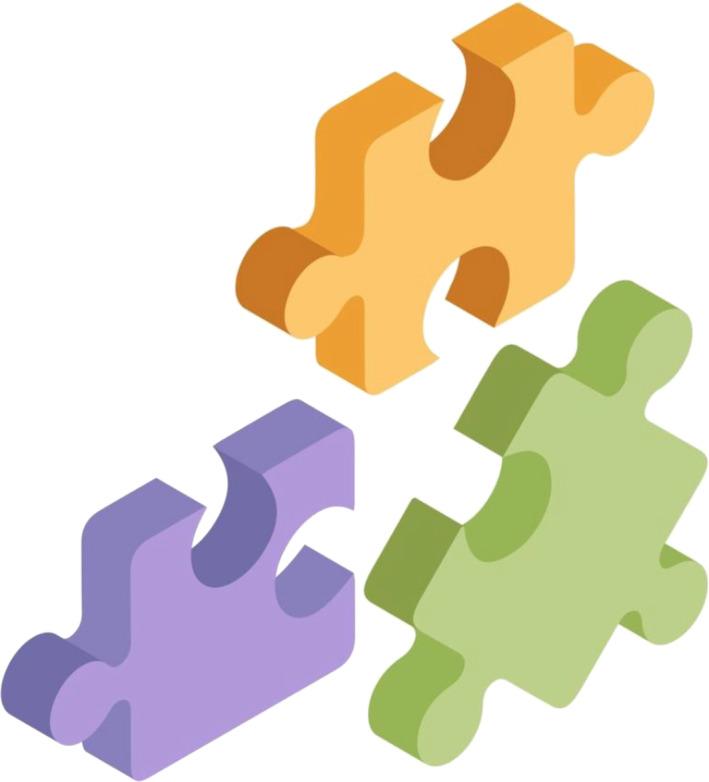

Cell communication and signaling are two major regulatory biological pathways involved in controlling the harmonious interaction of tissues and organs from early normal development to the late stages of the life cycle. Dysregulation of these pathways can occur at any level and have deleterious consequences, ranging from visible aberrant behavioral or physical manifestations to pathological medical conditions resulting from the lack of immunological defense, abnormal signal emission, transmission, and/or reception and interpretation.

Discovered two decades after the identification of three genes (Cyr61, Ctgf, and Nov, also known as Cell Communication Network (CCN) 1–3) encoding structurally very similar proteins, with a variety of different biological properties, these regulatory factors were the prototypes of a larger family of CCN factors. This family includes many previously described multifactorial signaling families involved in cellular signaling and communication (Figure 1).

**FIGURE 1:**
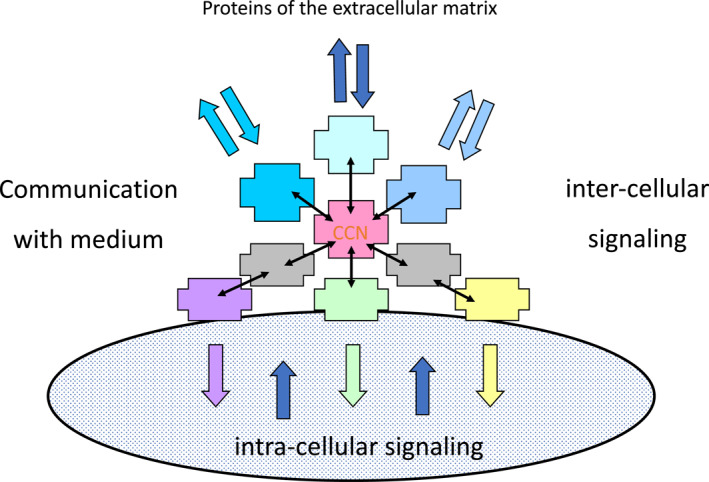
The realm of cell communication network interactions.

As one of the three pioneers in this field, Bernard Perbal believed that we should further enhance the scientific progress by collaborating with groups working on these proteins. In 1999, he proposed a meeting in Saint‐Malo, France, to share our views and ideas on the future development of this growing field. The diverse and engaging presentations at the event laid the foundation for international collaborations and productive interactions among groups studying these new signaling factors. This initiative also paved the way for the establishment of the nonprofit International CCN Society.^1^

Studies performed by different groups on CCN proteins over the last 30 years have highlighted the need for a more comprehensive approach to fully understand the biological activities regulated by these proteins. For example, research carried out in different independent laboratories has highlighted the numerous spatial interactions of CCN proteins with other signaling factors. In particular, in the case of CCN3, its biological activities were found to be regulated in a spatio‐temporal manner through multiple interactions with signaling partners located in intra‐ and extracellular compartments.^2^ In addition to its function as a secreted matricellular regulator, CCN3 was found to be present in the nucleus of undifferentiated normal cells and highly proliferative cancer cells, where it physically interacts with the Rpb7 regulatory subunit of eukaryotic RNA polymerase ^3^ and with IL‐33.^4^ Further studies established that the nuclear CCN3 protein can act as trans‐repressor of transcription,^5^ suggesting that it may act as a co‐factor in multi‐protein complexes that govern the regulation of transcription.^4^ Additionally, nuclear CCN3 has been shown to disrupt cell cycle progression,^5^ and was detected by immunogold staining at the nuclear pore of adenocarcinoma cells NCI‐HR295R.^6^

Unfortunately, a full exploration of these observations required expertise and resources that this group and others in the CCN field did not have access to. Due to the extensive association of CCN proteins with important biological signaling pathways, it has been suggested that they represent both a central communication and coordination network.^7^, ^8^, ^9^

At the 2019 Niagara CCN workshop, Bernard Perbal suggested expanding the theme of both ICCNS and of the Journal of Cell Communication and Signaling (JCCS) in a practical way to attract researchers willing to contribute their expertise and pursue collaborative projects with CCN teams. The idea was to create a nonprofit association called ARBIOCOM (short for Association for Research on Biosignaling and Communication), officially recognized in 2020. The main goal of this association is to bring different societies (academic, pharmaceutical, medical, and others) and journals (JCCS, Biomedicines, Cell, International Journal of Molecular Sciences, Fibrosis, Cell Communication and Signaling, Metabolism and Target Organ Damage, and others) together in the future, with a focus on basic molecular and spatial biology biosignaling and communication, cell biology, metabolomics, biochemistry, development biology and many others, forming a multidisciplinary scientific organization (Figure 2).

**FIGURE 2:**
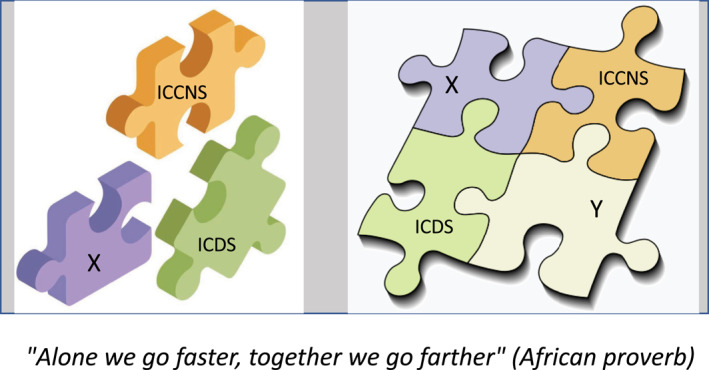
Functional interactions of participating societies within the Association for research on biosignaling and communication framework. This association will bring together scientists from academic, pharmaceutical, medical, and various other disciplines. It will include the International Cell Death Society, the ICCNS, and others indicated by X and Y.

This “melting pot” could encompass various processes such as cellular signal transduction, intercellular communication in the microenvironment, long‐distance communication involving soluble circulating factors such as hormones and extracellular vesicles, and biological communication between cells and organs in response to bacterial and viral infections, as well as all aspects of cellular and organ metabolism.

The main objective of ARBIOCOM is to facilitate the meeting of members of the society at all levels, allowing them to share knowledge, expertise, information, and sophisticated advanced technologies on a common platform. In this collaborative network, the ICCNS would be just one of several other participants. Importantly, there would be no strings attached and no fees to be paid for any of the associated societies, journals, or academic partners. Moreover, all participating societies would retain their specificity and identity.

A previous brainstorming meeting on ARBIOCOM was organized by the ICCNS in Nice in 2022. The strong interest in this project expressed by some members of a preliminary Steering Committee encouraged us to broaden our vision and establish an official foundation for this organization.

An International Steering Committee composed of 10 members: Håvard Attramadal (Oslo, Norway), Brahim Chaqour (Pennsylvania, USA), Raymond B. Birge (New Jersey, USA), Bhudev C. Das (Noida, India), Milos Marinkovic (San Antonio, USA), Kathryn E. Meier (Spokane, USA), Vanja Pekovic‐Vaughan (Liverpool, UK), Bernard Perbal (Nice, France), Annick Perbal (Nice, France), and Ralf Weiskirchen (Aachen, Germany) met at the 12th International Workshop on the CCN Gene Family, which took place in Oslo from June 20–23.

It was suggested that this Committee should soon make proposals for the organization of this federation, and specifics regarding the participation of all societies in social and scientific events that might be of common interest (Figure 3).

**FIGURE 3:**
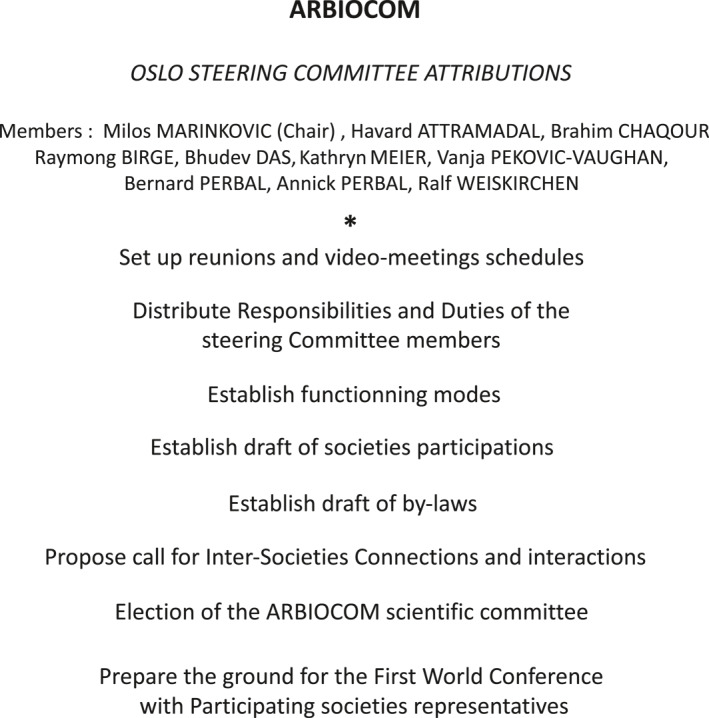
Oslo steering committee attributions.

Milos Marinkovic, who was invited to chair the session, is expected to present his proposals to the Steering Committee members regarding the participation of CCN society members in ARBIOCOM. The ARBIOCOM project will potentially include all societies willing to send students and seniors to our new meeting organization. Any suggestions for promoting links with other entities that may be interested in developing or sponsoring our joint endeavor are most welcome.

We were pleased to hear from our colleague Matta Csaba that the Hungarian Biochemical Society (HBS), which promotes the activities of Hungarian biochemists, molecular biologists and biotechnologists through non‐governmental activities,^10^ has expressed interest in starting negotiations. Raymond B. Birge and R. Weiskirchen also proposed to encourage the scientific journals that they actively manage to join ARBIOCOM.

With the help of all those interested in joining our proposal Annick and Bernard Perbal would be happy to host and launch our first ARBIOCOM meeting in 2025. This could be the occasion to celebrate the 25th anniversary of the ICCNS. The tentative title of our new ARBIOCOM meeting could be: “First World Conference on Cellular Signaling and Communication”. For information about the ARBIOCOM, please contact Annick Perbal or Bernard Perbal at “info@arbiocom.org”.

## AUTHOR CONTRIBUTIONS

Conceptualization, Perbal Bernard; writing – original draft preparation, Perbal Bernard; writing – review and editing, Perbal Bernard and Ralf Weiskirchen. All authors have read and agreed to the published version of the manuscript.

## CONFLICT OF INTEREST STATEMENT

B.P. is the President of the CCN Society, a nonprofit association. Furthermore, he is the Editor‐in‐Chief JCCS that is the official journal of the International CCN Society. R.W. declares that he has no known competing financial interests or personal relationships that could have appeared to influence the work reported in this paper.

## ETHICS STATEMENT

Not applicable.

## Data Availability

Not applicable; no new data was created or analyzed in this study.
